# Development of an implementation plan for a school-based multimodal approach for depression and suicide prevention in adolescents

**DOI:** 10.3389/fpubh.2024.1386031

**Published:** 2024-05-10

**Authors:** Kristel Jenniskens, Sanne Rasing, Arne Popma, Daan Creemers, Chaimae Ghalit, Leonie van Vuuren, Saskia Mérelle, Jan Spijker, Femke van Nassau

**Affiliations:** ^1^GGZ Oost Brabant, Boekel, Netherlands; ^2^113 Suicide Prevention, Amsterdam, Netherlands; ^3^Behavioural Science Institute, Radboud University, Nijmegen, Netherlands; ^4^Child and Adolescent Psychiatry & Psychosocial Care, Amsterdam, Netherlands; ^5^Department of Public and Occupational Health, Amsterdam UMC Location Vrije Universiteit Amsterdam, Amsterdam, Netherlands; ^6^Amsterdam Public Health Research Institute, Health Behaviors & Chronic Diseases, Amsterdam, Netherlands; ^7^Pro Persona, Nijmegen, Netherlands

**Keywords:** implementation mapping, implementation, adolescents, prevention, depression, suicide

## Abstract

Strong Teens and Resilient Minds (STORM) is a multimodal, school-based approach for depression and suicide prevention in adolescents that is currently implemented in a region in the Netherlands. The STORM approach will be implemented in new regions in the coming years. This study used the implementation mapping protocol to report on the development of the STORM implementation plan. First, a needs assessment was conducted through semi-structured interviews with stakeholders and brainstorming sessions with regional programme leaders in the two regions that started implementing STORM in 2023. This led to the identification of six main barriers to implementation: high level of demands for schools, insufficient understanding of the programme content, insufficient network collaboration, no perceived relative advantage of STORM by stakeholders, lack of attention to sustainability, and high work pressure. Second, performance and change objectives were formulated based on these barriers. For example, a performance objective for potential providers was that they felt supported by STORM. Third, implementation strategies were selected from theory and translated into practical applications through brainstorming sessions with programme leaders. The following strategies were included in the implementation plan: collaborate with similar initiatives within the region, free up time for STORM tasks, tailor strategies, identify and prepare STORM champions, and promote network weaving. Last, a plan to evaluate the implementation of STORM and the application of the STORM implementation plan was formulated. Planned evaluation research will provide more insight into the usefulness and impact of the STORM implementation plan.

## Introduction

Globally, depression and suicide prevalence in adolescents is high and appears to be increasing (1–8). Adolescent depression is associated with poor social well-being, poor school attendance, failure to complete secondary school, depression recurrence, and the onset of other psychiatric disorders (9–12). Moreover, suicide is the fourth leading cause of death among adolescents aged 15–29 worldwide (13). This stresses the need to implement evidence-based depression and suicide prevention programmes.

Educational settings offer opportunities to reach a large number of adolescents, since most adolescents attend school. Several review studies have found small effects and moderate effects on students’ mental health for universal and indicated school-based depression prevention interventions, respectively (14–17). School-based suicide prevention interventions have shown small positive effects on suicidal ideation and behaviours (18, 19). Katz et al. (20) and Hofstra et al. (21) have suggested combining several interventions to further increase the efficacy of depression and suicide prevention.

Such an approach has been developed in the Netherlands and is called Strong Teens and Resilient Minds (STORM) (22, 23). Currently, the STORM approach consists of four interventions (22, 23): (1) universal prevention through mental health lessons in schools, (2) a gatekeeper training (GKT) for school personnel to create a support network around adolescents, (3) early detection of depressive symptoms and suicidality and further assessment and referral when needed, and (4) Op Volle Kracht (OVK, which translates to “at full force”), an indicated depression prevention intervention based on cognitive behavioural group therapy. The STORM approach is science-based, and several programme components have been found to be effective (22, 23): The GKT has been found effective at increasing knowledge of suicide prevention and confidence to discuss suicidality (24). The OVK training has been found effective at reducing depressive symptoms in adolescent (25, 26).

Despite the existing evidence on the effectiveness of interventions for mental health promotion, prevention, and treatment, most people affected by mental health problems do not receive appropriate intervention (27). Therefore, scaling up effective prevention approaches is warranted. As part of the Dutch National Agenda Suicide Prevention 2021–2025 (28), which states national-level goals and activities in the context of suicide prevention, STORM will be scaled up to a national level. STORM is currently implemented in one region in the Netherlands that has about 250,000 inhabitants. Several new Dutch regions will be financially supported to also implement the approach in the coming years. Higher levels of implementation in various implementation outcomes, such as fidelity or dose, are related to better programme outcomes (29–31). This requires applying strategies that fit the context of new user settings (32). Therefore, developing an implementation plan in collaboration with stakeholders is essential to enhance the level of implementation and the potential programme outcomes.

The current study reports on the development of an implementation plan for STORM using the implementation mapping protocol, a systematic approach to developing an implementation plan by combining theory and co-creation with stakeholders in practice (32). Studies reporting on the development of an implementation plan for school-based mental health approaches in preparation for implementation are scarce. While we studied the example case of STORM, our approach to identifying these strategies and our outcomes could inform other school-based mental health approaches as well. This case is of particular interest to others, because of the complexity of STORM considering the multiple components, and because many stakeholders are involved in providing and implementing the approach.

## Methods

The current study used a qualitative case study design to develop an implementation plan for STORM that was co-created with stakeholders in practice, and was guided by thematic analysis (33). The report followed the Standards for Reporting Qualitative Research formulated by O’Brien et al. (34), which was filled in and included in Supplementary File 1. All participants in this study signed an informed consent form before participation. This study was approved by the Ethics Commission Social Sciences of Radboud University, approval number ECSW-LT-2023-2-2-33415.

### The STORM approach

First, mental health lessons are offered by mental healthcare professionals in schools to improve mental health literacy. Second, schoolteachers can undergo GKT, through which they learn to identify adolescents who show signs of suicidal behaviours and how to respond to those students. Third, a screening of students’ depression and suicide risk is conducted by the Public Health Service (PHS, in Dutch: GGD) using the Childhood Depression Inventory 2 (35) and the Questionnaire Assessing Suicide and Self Injury (36). Students identified as at risk for suicide are seen within 48 h by Child and Youth Health (CYH) professionals from the PHS for further assessment and referral, if necessary. Students with elevated depressive symptoms based on the Child Depression Inventory 2 are offered the indicated depression prevention intervention called OVK, which is based on cognitive behavioural group therapy (23). This intervention is usually provided by a duo of a care professionals within the school and a care professional in the youth care domain.

An integral part of STORM is collaboration within the network of care and education for adolescents (22, 23, 37). There are four main partners in this network: secondary and vocational schools, municipalities, PHS, and mental health professionals. Secondary and vocational schools are the settings for all interventions that are part of STORM (23). Within a region, these schools collaborate with municipalities in educational partnerships in supporting and caring for youth (38). Also, municipalities financially facilitate the implementation of STORM in practice (23). While regions can apply for a start-up budget through the Dutch National Agenda Suicide Prevention 2021–2025 subsidised by the Ministry of Health, Welfare, and Sports (28), municipalities still have to be involved for sustained financing after 2025. A team of mental health professionals provides consultation, training and personnel for carrying out the interventions (23).

**Figure 1:**
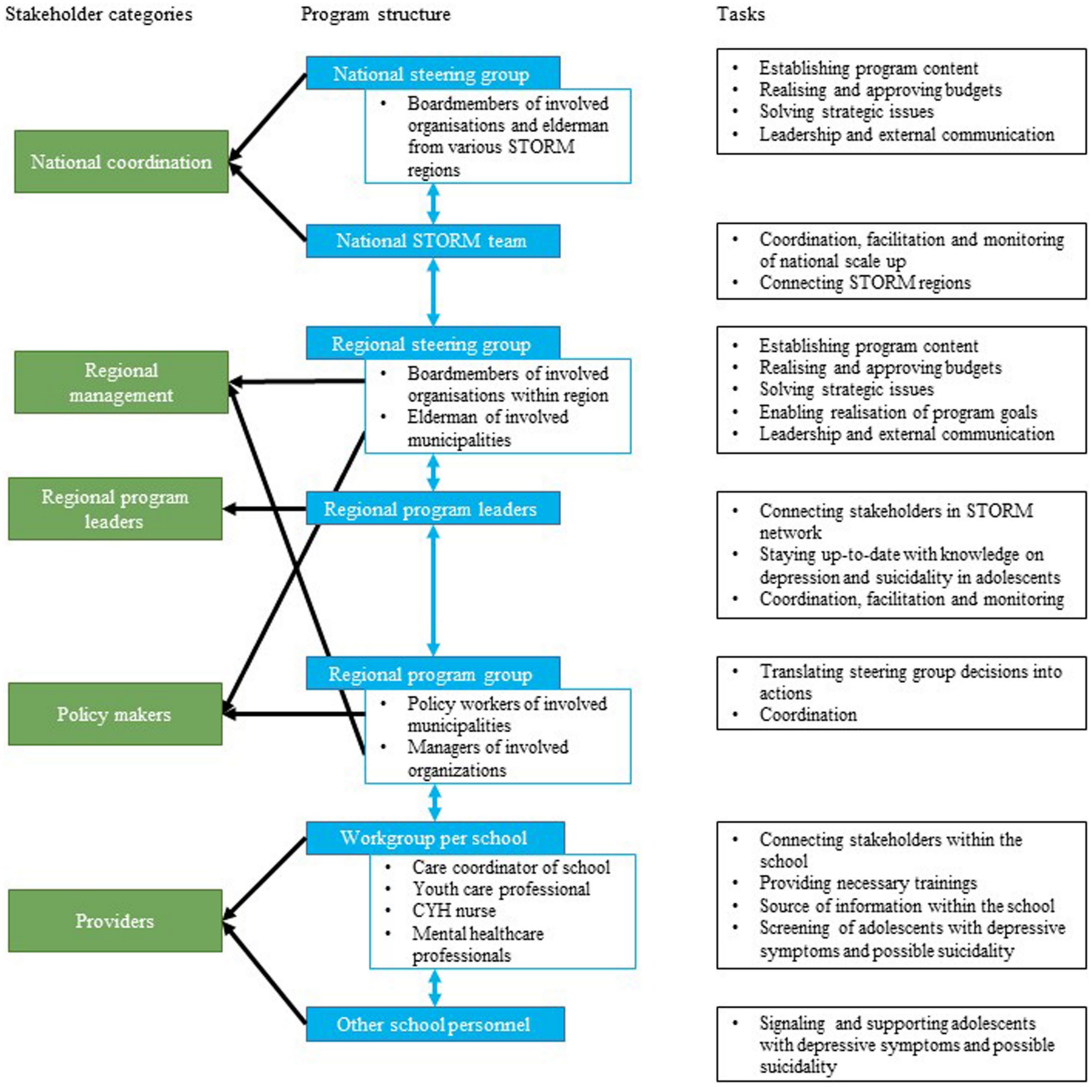
Overview of regional STORM programme structure.

An overview of the regional STORM programme structure, including the tasks of each partner, is provided in the second and third columns of Figure 1. Stakeholders from education, the PHS, mental health services, and municipalities collaborate in each part of the programme structure. For the current study, we defined four stakeholder categories: regional management, regional programme leaders, policymakers, and service providers. These are also indicated in the first column of Figure 1.

### National scaling up of STORM

As part of the Dutch National Agenda Suicide Prevention 2021–2025 (28), STORM is being scaled-up to new regions in the Netherlands between 2021 and 2025. Interested regions could apply for a start-up implementation budget. The first two regions to receive this budget were selected in December 2022 and started implementing STORM in the academic year of 2023–2024 (39). To apply for the budget, the regions had to prepare for implementation and had thus already initiated several implementation strategies before the start of the current study. Furthermore, existing STORM regions have already introduced several implementation strategies in recent years. These strategies have already been formulated and provided to the new regions. An overview of the existing implementation strategies can be found in Supplementary File 2. The current study seeked to identify additional strategies from the literature that can help to overcome implementation barriers.

### Theoretical background

The tasks of implementation mapping (IM) described by Fernandez et al. (32) offer a systematic approach to developing an implementation plan by combining theory and co-creation with stakeholders in practice. IM has previously helped to identify implementation strategies for various preventive interventions and programmes (40–46). The five tasks of IM are the following: (1) conduct an implementation needs assessment to identify barriers and facilitators for implementation, (2) identify adoption and implementation outcomes, performance objectives, and change objectives, (3) select theoretical methods and design implementation strategies, (4) produce implementation protocols and materials, and (5) evaluate implementation outcomes (32).

We used the Consolidated Framework for Implementation Research (CFIR) from Damschroder et al. (47) to identify barriers to and facilitators for implementation in Task 1. The CFIR describes constructs in five domains to consider as potential barriers or facilitators. First, the innovation domain, which includes constructs related to the innovation being implemented. Second is the inner setting into which the innovation is implemented. Third is the outer setting within which the inner setting exists. The fourth domain concerns individuals and pertains to the roles and characteristics of individuals involved in the innovation being implemented. The last domain implementation process consists of constructs related to the activities and strategies used to implement the innovation (47).

For the selection of theoretical implementation strategies in Task 3, Powell, Waltz (48) compiled a list of 73 implementation strategies based on the results of the Expert Recommendations for Implementing Change (ERIC) study. Recently, this compilation has been adapted to improve its utility in educational settings in the School Implementation Strategies, Translating ERIC Resources (SISTER) Project (49). This project resulted in a list of 75 school-adapted implementation strategies. Both the ERIC and SISTER compilations guided the selection of strategies in this study.

**Figure 2:**
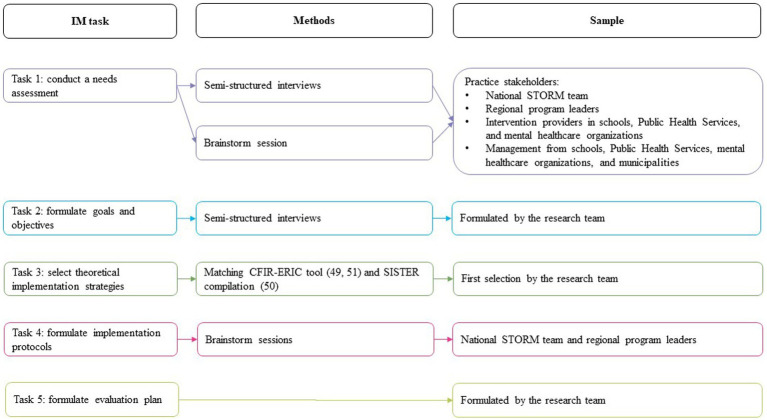
Overview of procedures and sample per IM task.

Below, we specify our study procedures in terms of sample and recruitment, data collection, and data analysis conducted for each of the five IM tasks. An overview of our procedures for each task is described in Figure 2.

### Task 1: conduct a needs assessment

The needs assessment helps to identify important actors and potential barriers to and facilitators for implementation (32). For this, we conducted semi-structured interviews of approximately half an hour in February and March 2023. Study participants were selected from the region that has already implemented STORM (region 1) and two regions that were planning to implement STORM (regions 2 and 3) using purposive snowball sampling. First, we invited the national STORM team and regional programme leaders (*n* = 6) for interviews. Next, they helped to identify and contact other relevant stakeholders within the regions. We aimed to represent all stakeholder groups, and reached out to management and intervention providers from schools (*n* = 9), the PHS (*n* = 5), mental health organisations (*n* = 5), and municipalities (*n* = 4). Additionally, we invited mentors from secondary schools that had already implemented STORM (*n* = 2) for interviews, because mentors from secondary schools in the new regions had not yet been informed about the STORM approach. Participants were included until data saturation was reached, which meant that two researchers (KJ and CG) agreed that the last two interviews did not lead to new information. Researchers met with the national coordinators and regional programme leaders prior to the interviews to discuss which other stakeholders to include in the study. Researchers did not meet with any of the other participants prior to the interviews. The researchers’ characteristics did not influence the research questions, approach, methods, results, or transferability.

The interviews followed an interview guide based on the updated CIFR (47). The topics included STORM characteristics (example question: what is your perspective on STORM?) and barriers and facilitators (example question: what are things you think could complicate the implementation of STORM?), as well as the sub-topics Outer Setting, Inner Setting, Individuals, and Implementation Process. The full topic list is added in Supplementary File 3. Interviews were conducted by two researchers (KJ & CG) and audio-recorded. KJ was a PhD student at the time of the study with previous experience in conducting and analysing qualitative research. CG was a bachelor student and intern at the time of the study with no previous experience in qualitative research. The recordings were transcribed verbatim. After the interviews, a short summary was sent to the participants for verification.

To draw up a codebook, three researchers (KJ, CG, and FN) analysed six of the 20 interviews using open coding in Atlas.ti. FN (PhD) has previous experience in conducting and analysing qualitative research. Two researchers (KJ & FN) ordered the codes under the five major domains of the CFIR framework (47) and then combined them into overarching codes using axial coding. The complete codebook can be found in Supplementary File 4. Next, two researchers (KJ & CG) separately coded three transcripts, after which the coding was compared and variations in coding were discussed until both researchers agreed. Subsequently, all interview transcripts were analysed using deductive coding. Finally, the researchers analysed the coded data to identify barriers to and facilitators for the implementation of STORM in new regions.

Two researchers (KJ and FN) presented the identified barriers to four programme leaders from regions 2 and 3 and two stakeholders who had been involved in the implementation of STORM in region 1 during a brainstorming session in May 2023. We asked these participants to indicate, on a scale from 1 to 5 per barrier, whether they thought a barrier required immediate action or not using Mentimeter. The results of this brainstorming are available in Supplementary File 5 (in Dutch). Barriers that were scored higher than 3.5 were selected, while barriers that were scored lower than 2.5 were not. For barriers that were scored between 2.5 and 3.5, a group discussion determined whether the barrier was selected.

### Tasks 2, 3, and 4: formulate goals, objectives, implementation strategies, and implementation protocols

We formulated performance objectives based on the most important barriers identified in Task 1. For each performance objective, we formulated change objectives across five determinants based on the example of Kang and Foster (46): knowledge, awareness, skills, outcome expectancy, and self-efficacy. We chose this example, because it was the most complete objectives matrix we found.

Next, we selected theoretical implementation strategies to achieve the change objectives. First, for determinants that match the first-version CFIR constructs (47), strategies were identified using the CFIR-ERIC tool (48, 50). These strategies were then compared to the adapted compilation of ERIC implementation strategies for school-based implementation, SISTER (49), to identify strategies that are suitable for school-based implementation projects. Then, for determinants that did not fit the first-version CFIR constructs, suitable strategies were selected from the SISTER strategies (49).

A second round of brainstorming sessions was hosted in May 2023, one session with two national coordinators and one with four regional programme leaders. In both sessions, the main author (KJ) presented identified strategies for the selected barriers. Participants first discussed which of the identified strategies overlapped with existing implementation strategies. For the remaining strategies, participants discussed the extent to which they were realistic and relevant for practice. The implementation strategies that were considered both realistic and relevant for practice by national coordinators and regional programme leaders were selected for the implementation plan. If participants did not reach consensus about how realistic or relevant a strategy would be, the main author (KJ) made the final decision to include or exclude the strategy. A detailed description of our selection process can be found in Supplementary File 2. Next, the selected strategies were translated into practical applications in collaboration with the programme leaders from regions 2 and 3. The applications were reported following the recommendations for reporting implementation strategies by Proctor et al. (51). The implementation strategies found in the current study were added to the STORM implementation guide developed by the national STORM team. Besides implementation strategies, this document contains a detailed description of the programme components and programme structure of STORM. All future STORM regions will be offered this guide to aid their implementation efforts.

### Task 5: develop an evaluation plan

Based on the implementation plan, KJ and FN developed a plan to evaluate the implementation of STORM, as well as the application of the implementation plan in practice.

## Results

### Task 1

Twenty stakeholders were interviewed. Stakeholders were included until data saturation was reached. Their characteristics are presented in Table 1. Most participants had a coordinating role in the project, followed by providers, management, and policymakers. The number of participants per organisation type were spread evenly, except for municipalities, which were represented by only two participants.

**Table 1:** Participant characteristics.

Category	Group	Number of participants
Region	1	2
	2	7
	3	9
	National	2
Project role	Coordination	8
	Provider	7
	Management and policy	5
Organisation	Mental health services	4
	Municipality	2
	Public Health Services (PHS)	4
	School	5
	Other	5

We identified 21 barriers to and 13 facilitators for the implementation of STORM in new regions. An overview of all identified barriers and facilitators can be found in Supplementary File 6. These determinants include CFIR constructs and barriers that did not match the CFIR constructs. In the first brainstorming session with programme leaders, five barriers were selected for which implementation strategies should be identified. An overview of these barriers is presented in Table 2.

**Table 2:** Barrier descriptions.

Barrier	Description	Quotes
High demand for schools	Participants mentioned that schools are often seen as the ideal setting for any kind of preventive intervention, leading to high societal demands for schools.	*‘Teachers have to be good at math, Dutch language, and so on, but they are not healthcare professionals.’* – Employee municipality 1, region 3 *“Why should teachers do that? Let them work on education and their relation with their students.”* – Alderman 1, region 3
High work pressure	Participants reported high work pressure in education and healthcare.	*‘The whole society is impacted by the [shortages on] the labour market […], that also applies to the healthcare field.’* – Alderman 1, region 3 *‘All schools are dealing with staff shortages.’* – National coordinator 1 *‘There’s a shortage in Child and Youth Healthcare physicians, so how are you going to do [STORM]?’* – School principal 1, region 3
Insufficient understanding of program	Participants indicated that stakeholders did not sufficiently understand the content of STORM and what it means in practice.	*‘I do think it’s very complex, because I’ve read it a few times now, and still I think ‘huh? What should happen exactly?”* – Employee municipality 1, region 3
Insufficient network collaboration	Participants reported insufficient collaboration between organisations within the network, especially between schools and mental healthcare organisations. Programme leaders considered this the most relevant barrier.	*‘In some municipalities, schools are more eager to collaborate with us than in other municipalities.’* – Manager Public Health Services 1, region 2 *‘We do not know from each other [who has which role]. Mapping the whole network seems important to me.’ –* Programme leader 3, region 2 *‘If students need something externally, we have to deal with a lot of institutions. Before we had a regular consultation with someone from mental healthcare. Unfortunately, that has been cut back.’* – School therapist 1, region 2
Lack of attention for sustainability	During the brainstorm sessions, programme leaders discussed that future sustainment of STORM did not receive enough attention yet.	
No perceived relative advantage	Participants thought that not all stakeholders see an advantage of STORM compared to other interventions.	*‘Mental health organizations say: ‘we already do something similar to Op Volle Kracht. We have just been trained in this last year and now we should throw that overboard.”* – National coordinator 1 *‘Municipalities have to make all kinds of choices. Prevention can be a difficult one, because you cannot say “again five adolescents who did not commit suicide.”’* – National coordinator 2 *“For [the Public Health Services] it was a requirement to keep using our own, broader [health check] questionnaire. Otherwise you have questionnaire for every problem, that does not have my preference.’* – Manager Public Health Services 2, region 2

Multiple participants mentioned that schools are seen as the ideal setting for prevention, not only regarding mental health, but also for prevention of obesity or smoking. This leads to a high demand for schools. For example, one of the regional program leaders mentioned that *“many societal developments are occurring and often it’s the schools who have to solve it*.” Related to this, most participants recognised high work pressure in both education and healthcare as an important barrier for implementing a new approach. A school principal indicated that *“it is an important theme, but honestly I do not have the people, the time, and the money to properly implement STORM.”*

Another important issue raised by participants was that, at the time of the interviews, they, nor their colleagues, sufficiently understood the content of the STORM approach and what it means in practice. A school therapist mentioned, for example, that they *“still need to receive a lot of information*.” Moreover, participants thought that not all stakeholders saw added value in STORM compared to other interventions. A national coordinator indicated that *“[organizations] struggle to de-implement [what they were already doing] to implement of STORM.”*

It was also noted by some participants that network collaboration required improvement, especially between schools and mental healthcare services. A manager in mental healthcare mentioned that *“education sometimes complains: ‘[mental healthcare organizations] do some test, but they never refer back to us’,”* while a school therapist mentions that *“collaboration [with mental healthcare organizations] does not exist in our school.”*

Additional to the interview results, lack of attention to the sustainability of STORM in the current implementation efforts was identified as a barrier during the brainstorming sessions. Programme leaders felt that long-term sustainability was not receiving enough attention yet from stakeholders involved.

### Task 2

For each barrier selected in Task 1, we formulated performance and change objectives. The performance and change objectives are listed per stakeholder category in Table 3. For example, a performance objective for programme leaders related to the barrier ‘insufficient network collaboration’ and was that they should stimulate the development of sustainable partnerships between involved organisations. Change objectives for this performance objective were formulated under skills (i.e., able to connect organisations within the STORM network) and self-efficacy (i.e., are confident that they are able to connect organisations within the STORM network).

**Table 3:** Matrix of change.

Barrier	Performance objective	Change objective				
		Knowledge	Awareness	Skills	Outcome expectancy	Self-efficacy
Programme leaders						
Insufficient network collaboration	**R1** Stimulate development of sustainable partnerships between involved organisations.			**R1a** Are able to connect organisations within the STORM network.		**R1b** Are confident that they are able to connect organisations within the STORM network.
Lack of attention for sustainability	**R2** Ensure sustainment of STORM after 2025.		**R2a** Are aware of willingness of involved parties within the region to continue working with STORM.	**R2b** Are able to keep parties involved and willing to continue with STORM.		**R2c** Are confident that they are able to ensure the sustainment of STORM after 2025.
Regional management						
High demands schools	**M1** Feel supported by STORM.				**M1a** Expect that STORM can help to support adolescents struggling with depression and/or suicidality.	
Insufficient understanding of programme content	**M2** Provide clear information about STORM to involved employees.	**M2a** Understand what STORM entails both in theory and in practice.				**M2b** Are confident that they have sufficient knowledge of STORM to inform stakeholders within their organisations.
Insufficient network collaboration	**M3** Develop sustainable partnerships with other organisations involved in STORM.		**M3a** Are aware of other organisations that are part of the STORM network.		**M3b** Expect that collaborating with other organisations leads to better implementation outcomes.	
No perceived relative advantage	**M4** Perceive added value of STORM compared to other approaches.	**M4a** Understand what STORM entails both in theory and in practice.			**M4b** Expect that STORM is beneficial for the wellbeing of adolescents compared to other approaches.	
Lack of attention for sustainability	**M5** Embed STORM within their organisation.		**M5a** Are aware that embedding STORM is needed for sustainment of STORM over time.		**M5b** Expect that embedding STORM will contribute to sustainment over time.	
High work pressure	**M6** Minimise workload for employees due to STORM.		**M6a** Are aware of (potential) extra workload for employees to fulfil their role in STORM.		**M6b** Expect that minimising workload leads to better implementation outcomes.	
Service providers						
High demands schools	**D1** Feel supported by STORM.				**D1a** Expect that STORM can help them in providing support for adolescents struggling with depression and/or suicidality.	
Insufficient network collaboration	**D2** Collaborate with others in the STORM network.		**D2a** Are aware of other stakeholders that are part of the STORM network.		**D2b** Expect that collaborating with other stakeholders leads to better implementation outcomes.	
No perceived relative advantage	**D3** Perceive benefit of STORM compared to other approaches for depression and suicide prevention in adolescents.	**D3a** Understand what STORM entails both in theory and in practice.			**D3b** Expect that STORM is beneficial for the wellbeing of adolescents compared to other programmes.	
Lack of attention for sustainability	**D4** Embed STORM within their daily practice.		**D4a** Are aware of the advantages of STORM for the wellbeing of adolescents.	**D4b** Are able to embed STORM within their daily practice.	**D4c** Expect that sustaining STORM will lead to better outcomes in adolescents.	
High work pressure	**D5** Have enough time to fulfil their role in STORM.				**D5a** Expect that they can fulfil their role in STORM within their work schedule.	**D5b** Are confident that they can fulfil their role in STORM.
Policy makers						
Insufficient network collaboration	**P1** Develop sustainable partnerships with other organisations involved in STORM.		**P1a** Are aware of other organisations that are part of the STORM network.		**P1b** Expect that collaborating with other organisations leads to better implementation outcomes.	
No perceived relative advantage	**P2** Perceive added value of STORM compared to other approaches for depression and suicide prevention in adolescents.	**P2a** Understand what STORM entails both in theory and in practice.			**P2b** Expect that STORM is beneficial for the wellbeing of adolescents compared to other programmes.	
Lack of attention for sustainability	**P3** Invest in the sustainment of STORM within their region.		**P3a** Are aware of the advantages of STORM for the wellbeing of adolescents.		**P3b** Expect that sustaining STORM will lead to better outcomes in adolescents.	

Not all barriers were relevant for each stakeholder category. ‘Partnerships and connections’ and ‘Sustainability’ were relevant for all categories, because all stakeholders are part of the STORM network, and the sustainable implementation of STORM should be achieved for all categories. ‘Relative advantage’ was not relevant for the programme leaders, because this is not a barrier for this stakeholder category based on the interviews. Since only the schools, the PHS, and mental healthcare organisations are involved in implementing STORM, the barriers ‘High demand for schools’ and ‘High work pressure’ were only relevant to these stakeholder categories. Finally, ‘Insufficient understanding of programme content’ was regarded as a relevant barrier for management only, because management has the final decision to participate in STORM, and thus needs to be well informed of the content.

### Tasks 3 and 4

To address the performance and change objectives formulated in Task 2, 14 implementation strategies were selected using the CFIR-ERIC tool and SISTER that matched the barriers identified (49, 50). Using the second round of brainstorming sessions with national coordinators and regional programme leaders, five implementation strategies were deemed relevant and realistic for practice: ‘pruning competing initiatives’, ‘change/alter environment’, ‘tailor strategies’, ‘identify and prepare champions’, and ‘promote network weaving’ (49). We translated these to practical applications and report on the strategies in Table 4 following the recommendations of Proctor et al. (51). The first strategy described is ‘collaborate with similar initiatives’, in which the idea is that regional program leaders actively identify other mental health school-based initiatives that are (being) implemented in their region, and look for ways to collaborate in the implementation process. The goal is to relieve the pressure on schools and minimise extra workload for service providers. Second is ‘free up time for STORM tasks’, in which organisation management allocate time to for implementing and executing STORM, while program leaders reserve budget to support organisations in doing so. Third is ‘tailor strategies’, meaning programme leaders adapt their communication style and message about STORM to the specific needs of various stakeholders, with the aim of improving adoption. Fourth is ‘identify and prepare STORM champions’, which entails both identifying and supporting individuals within involved organisations that are enthusiastic about the approach. The goal is to promote sustainment within those organisations through these individuals. Last is ‘regional network weaving’ through developing a social map of the organisations and individuals involved in STORM, and organising a joint kick-off session for those organisations and individuals.

**Table 4:** Implementation strategies

Implementation strategy	*Collaborate with similar initiatives*	*Free up time for STORM tasks*	*Tailor strategies*	*Identify and prepare STORM champions*	*Regional network weaving*
Definition	Collaboration with leaders from other school-based initiatives that are also aimed at improving the wellbeing of adolescents, especially if they also involve training of communication techniques for school personnel.	Extra time in the schedules of providers in STORM is freed up for STORM related tasks.	Strategies for getting organizations on board and for implementing STORM within organizations are tailored to the needs and perceptions.	Identification of individuals within organizations that are enthusiastic about STORM and willing to promote STORM within their organization, and supporting them.	Enabling network weaving between organizations involved in STORM on a regional level.
Actors	Programme leaders	Management, programme leaders	Programme leaders	Programme leaders	Programme leaders
Actions	Identify similar initiatives in (potential) STORM schools	Management allocates time in delivers' schedules for STORM tasks	Adapt their message about STORM to the target population	Identify champions of STORM within organizations	Develop a social map with contact details of all organizations and key figures in STORM within the region
	Approach leaders from these initiatives for collaboration	Programme leaders allocate regional STORM budget to support organizations for freeing up time for providers	Adapt their support style to the needs of organizations	Supporting champions of STORM by providing necessary information and materials and answering questions	Provide the social map to all stakeholders within the region
		Programme leaders explain how freeing up time for STORM tasks now leads to less time spend on counselling students later on			Revise social map annually
					Organize a joint STORM kick-off session for all involved stakeholders within the region
Action target	M1, M5, D1, D4	M5, M6, D4, D5	M3, M4, D3, D4, P2, P3	M4, M5, D3, D4, P2, P3	R1, R2, M3, M5, D2, P1, P3
Temporality	Continuous	Continuous	Continuous	Continuous	At the start of each school year
Dose	Not applicable	Not applicable	Not applicable	Not applicable	One social map, one half-day kick-off session
Implementation outcomes affected	Adoption, acceptability, feasibility, sustainability	Acceptability, feasibility, sustainability	Adoption, acceptability, penetration	Adoption, acceptability, appropriateness, sustainability	Feasibility, penetration, sustainability
Justification	Based on SISTER strategy ‘Pruning competing initiatives’ (50) and current practice.	Based on SISTER strategy ‘Change/alter environment’ (50) and current practice.	Based on SISTER strategy ‘Tailor strategies’ (50).	Based on SISTER strategy ‘Identify and prepare champions’ (49, 50).	Based on SISTER strategy ‘Promote network weaving’(50).

### Task 5

In the final task, we developed a plan to evaluate the implementation of STORM in the new regions, as well as the application of the implementation plan over the course of two academic years. We identified outcomes from the implementation outcomes defined by Proctor et al. (52) and process evaluation guidelines from Moore at al. (53) and Saunders et al. (54). In Table 5, we summarise the outcomes for the implementation of STORM, including definitions, and how and when the outcomes will be measured. Providers, regional management, and policymakers will be involved in the evaluation of the implementation process through a survey and interviews at multiple time points. The measurement instruments to be used in the survey comprise a shortened version of the Acceptability of Intervention Measure, Intervention Appropriateness Measure, and Feasibility of Intervention Measure from Weiner et al. (55), and the Normalisation Measure Development Questionnaire (56). In Table 6, we summarise the outcomes for the application of the implementation plan, including definitions of the outcomes, and how and when the outcomes will be measured. Programme leaders and programme groups (see Figure 1) will be involved in the evaluation through a checklist of implementation strategies and focus group sessions. Additionally, we will analyse the administrative data for both evaluations.

**Table 5:** Evaluation plan for the implementation of STORM.

Outcome	Definition	Data source						
		Providers, managers, policy makers						Administrative data
		Survey				Interviews		
		Autumn 2023	Spring 2024	Autumn 2024	Spring 2025	Spring 24	Spring 25	Continuous
Acceptability	Degree to which stakeholders perceive STORM as agreeable, palatable or satisfactory.	X	X	X	X	X	X	
Adoption	Stakeholders’ intention to employ STORM in practice and reasons for (not) employing STORM in practice.					X	X	
Appropriateness	Degree to which stakeholder perceive a fit between STORM and depression and suicide prevention in adolescents.	X	X	X	X	X	X	
Dose	Number of classes that received universal prevention; number of school personnel that followed Gatekeeper training; number of adolescents screened for depression and suicide risk; number of adolescents that participated in ‘Op Volle Kracht’.							X
Feasibility	Degree to which stakeholder think STORM can be successfully carried out within their organisation.	X	X	X	X	X	X	
Fidelity	Degree to which STORM is implemented in practice as intended.					X	X	
Implementation costs	Direct costs for execution of STORM in practice.							X
Normalisation	Degree to which STORM is normalised within everyday practice of providers.	X	X	X	X	X	X	

**Table 6:** Evaluation plan for the application of the implementation plan.

Outcome	Definition	Data source										
		Programme leaders and programme groups										Administrative data
		Checklist						Focus groups				
		Autumn 2023	Winter 2024	Spring 2024	Autumn 2024	Winter 2025	Spring 2025	Winter 2024	Spring 2024	Winter 2025	Spring 2025	Continuous
Acceptability	Degree to which stakeholders perceive implementation strategies as agreeable, palatable or satisfactory.							X	X	X	X	
Appropriateness	Degree to which stakeholder perceive implementation strategies as compatible for implementing STORM.	X	X	X	X	X	X	X	X	X	X	
Context	Determinants influencing the implementation of STORM.								X		X	
Dose	Application of implementation strategies.	X	X	X	X	X	X					
Feasibility	Degree to which stakeholder think the implementation strategies can be successfully carried out in their region.							X	X	X	X	
Fidelity	Degree to which implementation strategies are applied in practice as intended.	X	X	X	X	X	X	X	X	X	X	
Implementation costs	Costs related to the application of implementation strategies.	X	X	X	X	X	X					X
Reach	Characteristics of the setting and target population reached by STORM.							X		X		X

## Discussion

This study aimed to develop an implementation plan for a school-based approach to depression and suicide prevention. To our knowledge, this is the first study reporting on the development of an implementation plan for school-based mental health interventions. The IM tasks from Fernandez et al. (32) helped us to combine practical needs and perceptions with theoretical strategies. We identified six main barriers to implementation, on the basis of which we formulated performance and change objectives. We found five new implementation strategies to achieve these objectives. Lastly, we developed a plan to evaluate the implementation of STORM in new regions over the course of two school years.

One of the most relevant barriers to implementation of STORM that we found was limited network collaboration within regions, while network collaboration is an essential part of the STORM approach (22, 23, 37). A study into determinants for the screening and subsequent referral to the OVK revealed that even in the region where STORM has been implemented for years, collaboration between organisations involved in STORM is not optimal (57). This led us to the selection of several ERIC strategies categorised under ‘develop stakeholder interrelationships’ aimed at improving network collaboration (58). The implementation evaluation will determine whether these strategies indeed helped us increase network collaboration.

Some of the identified barriers were also some of the most frequently mentioned barriers for other school-based mental health interventions (59), including ‘insufficient understanding among stakeholders of programme content’ and ‘insufficient network collaboration’. However, costs and the availability of resources, which are often reported as barriers to implementation (59), were not identified as barriers in the current study. The fact that these factors were not discussed in any of the interviews is most likely due to the implementation budget that regions receive to implement STORM (28). However, this is only a start-up budget that can only be provided to a limited number of regions. Moreover, lack of funding or financial resources was identified as a barrier to sustaining school-based mental health interventions (60). Therefore, keeping track of implementation costs is relevant and has been included as an outcome in our evaluation plan.

We selected implementation strategies based on how realistic and relevant the STORM programme leaders thought they were. In a study by Lyon et al. (61), school-based consultants who provided social, emotional, and mental health services rated the feasibility and importance of all SISTER strategies. Most strategies we selected were also rated important in this study (61). Yet, we included some strategies which were rated low on feasibility in the study from Lyon et al. (61), including ‘collaboration with similar initiatives’, ‘use advisory boards and workgroups’, and ‘promote network weaving’, because they were considered realistic and relevant by the program leaders. These different perceptions might be explained by the difference in the stakeholders involved: we spoke with programme leaders, whereas Lyon et al. (61) consulted stakeholders within schools. However, these differences might also indicate the importance of context when considering the feasibility of an implementation strategy. Our evaluation of the implementation strategies should provide more insight into this difference.

The goal of the implementation plan developed in the current study is to improve the level of implementation of STORM in new regions in the Netherlands. We selected several implementation strategies that were found in the literature to have a positive effect on programme adoption and fidelity, including ‘conduct ongoing training’, ‘identify and prepare champions’, ‘use train-the-trainer strategies’, and ‘facilitation/problem solving’ (62). Still, knowledge about the mechanisms by which implementation strategies target their linked barriers, as well as about the effectiveness of most strategies, is lacking (62–64). Thus, while the IM approach helped us to select strategies that are likely to positively impact the implementation of STORM, our evaluation should confirm whether our selection was accurate.

### Strengths and limitations

A strength of the current study is that we systematically developed an implementation plan by following the tasks of IM (32). We did this in close collaboration with stakeholders who will implement STORM in practice, ensuring that the implementation plan matches the needs in practice. Additionally, we enhanced the credibility and transferability of our results through member checks, data and investigator triangulation, and sampling until we reached data saturation.

We recognise some limitations to our study as well. To begin, we mainly identified determinants related to the adoption and implementation of the intervention, and not to sustaining STORM over time. This is mostly likely because sustainability was not an explicit topic in our interviews and interviewees were in an early stage of pre-implementation. We discussed the lack of determinants with programme leaders and accordingly added a general determinant for sustainability. Furthermore, we reached out to multiple stakeholders and interviewed those who responded. Possibly, this led to selection bias if only participants with strong opinions about STORM, be these negative or positive, responded to our invitation. However, we asked participants to reflect on the perceptions of others in their field to minimise this bias.

## Recommendations

Building on our strengths and limitations, we first recommend following the tasks of IM when developing an implementation plan, as this helped us to systematically select appropriate strategies. Furthermore, it encourages close collaboration with practice, which we found to be very helpful for developing a plan that is both achievable and relevant for practice. In doing so, we recommend including sustainability in the needs assessment to identify determinants and strategies for sustainability within the implementation plan.

Second, we recommend consulting multiple sources for the selection of implementation strategies. We found it helpful to first use the CFIR-ERIC tool to get a first idea of possible strategies, and then compare them to the SISTER strategies to identify more suitable strategies for the school context. We recommend others developing an implementation plan to consult such strategy compilations for specific intervention settings, if available.

For new STORM regions, we recommend using this implementation plan as guidance rather than a prescription. Some strategies might prove not to be as relevant and/or feasible as we originally believed. The implementation plan could also be helpful for the implementation of other school-based mental health interventions as these might encounter similar barriers. However, tailoring the implementation strategies to the specific context for these interventions is warranted.

## Conclusion

In this study, we followed the tasks of IM, which helped us to develop a STORM implementation plan systematically and in collaboration with practice. The implementation plan offers guidance for new regions implementing STORM. Following the implementation plan could help to improve implementation outcomes and might even lead to better programme outcomes. Moreover, our approach and the strategies we identified could inform the implementation of other school-based mental health programmes, although we recommend tailoring our strategies to the specific context into which it will be implemented. Future research evaluating the implementation of STORM across the Netherlands will provide more insight into the usefulness of the implementation plan.

## Data availability statement

The raw data supporting the conclusions of this article will be made available by the authors, without undue reservation.

## Ethics statement

This study was approved by the Ethics Commission Social Sciences of Radboud University, approval number ECSW-LT-2023-2-2-33415. The studies were conducted in accordance with the local legislation and institutional requirements. The participants provided their written informed consent to participate in this study.

## Author contributions

KJ: Conceptualization, Formal analysis, Investigation, Methodology, Project administration, Visualization, Writing – original draft, Writing – review & editing. SR: Conceptualization, Supervision, Writing – review & editing, Writing – original draft. AP: Conceptualization, Supervision, Writing – review & editing, Writing – original draft. DC: Conceptualization, Supervision, Writing – review & editing, Writing – original draft. CG: Formal analysis, Investigation, Methodology, Writing – review & editing, Writing – original draft. LV: Investigation, Writing – review & editing, Writing – original draft. SM: Writing – review & editing, Writing – original draft. JS: Writing – review & editing, Writing – original draft. FN: Conceptualization, Formal analysis, Investigation, Methodology, Supervision, Writing – review & editing, Writing – original draft.
