# Supplementary material for: Development of an implementation plan for a school-based multimodal approach for depression and suicide prevention in adolescents
Source: Front Public Health. 2024 May 10;12:1386031. doi: 10.3389/fpubh.2024.1386031 (PMC11122015; doi:10.3389/fpubh.2024.1386031)
Supplement: Supplementary file 1 [file Data_Sheet_1.PDF]

Instructions

6

Ik vind dat we iets moeten doen met deze belemmeringen:

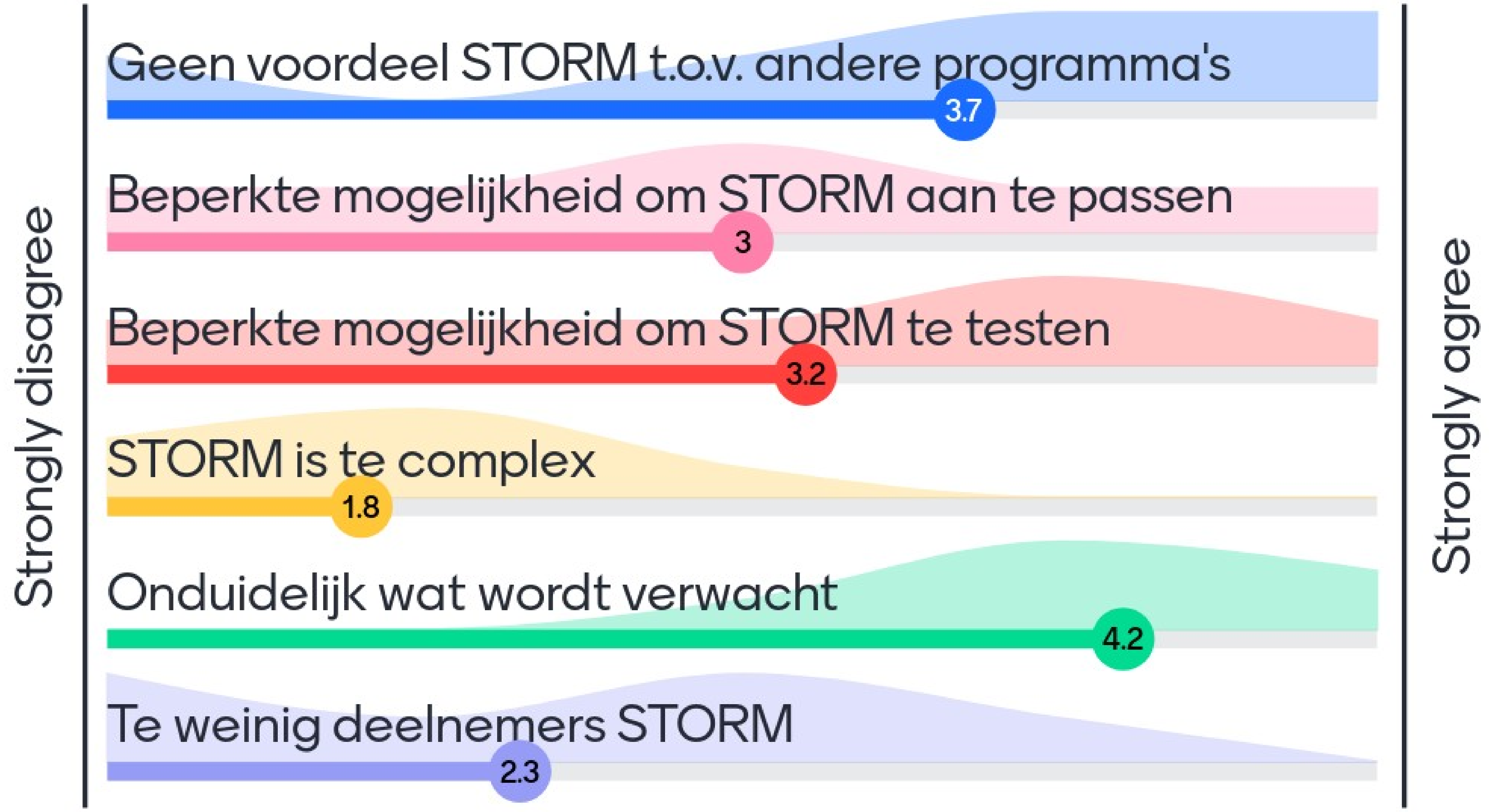

Mentimeter

6

Mentimeter

Ik vind dat we iets moeten doen met deze belemmeringen:

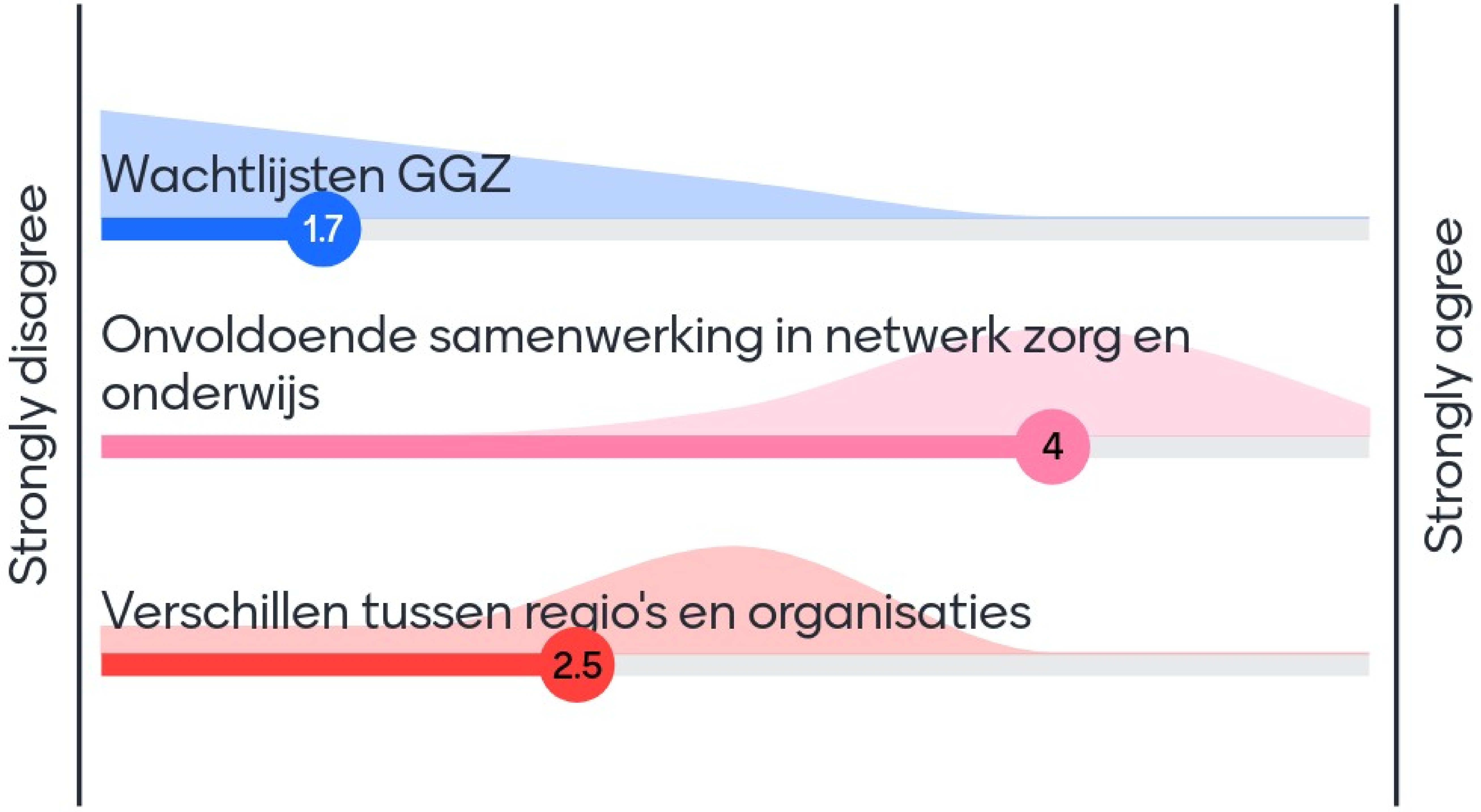

6

Mentimeter

Ik vind dat we iets moeten doen met deze belemmeringen:

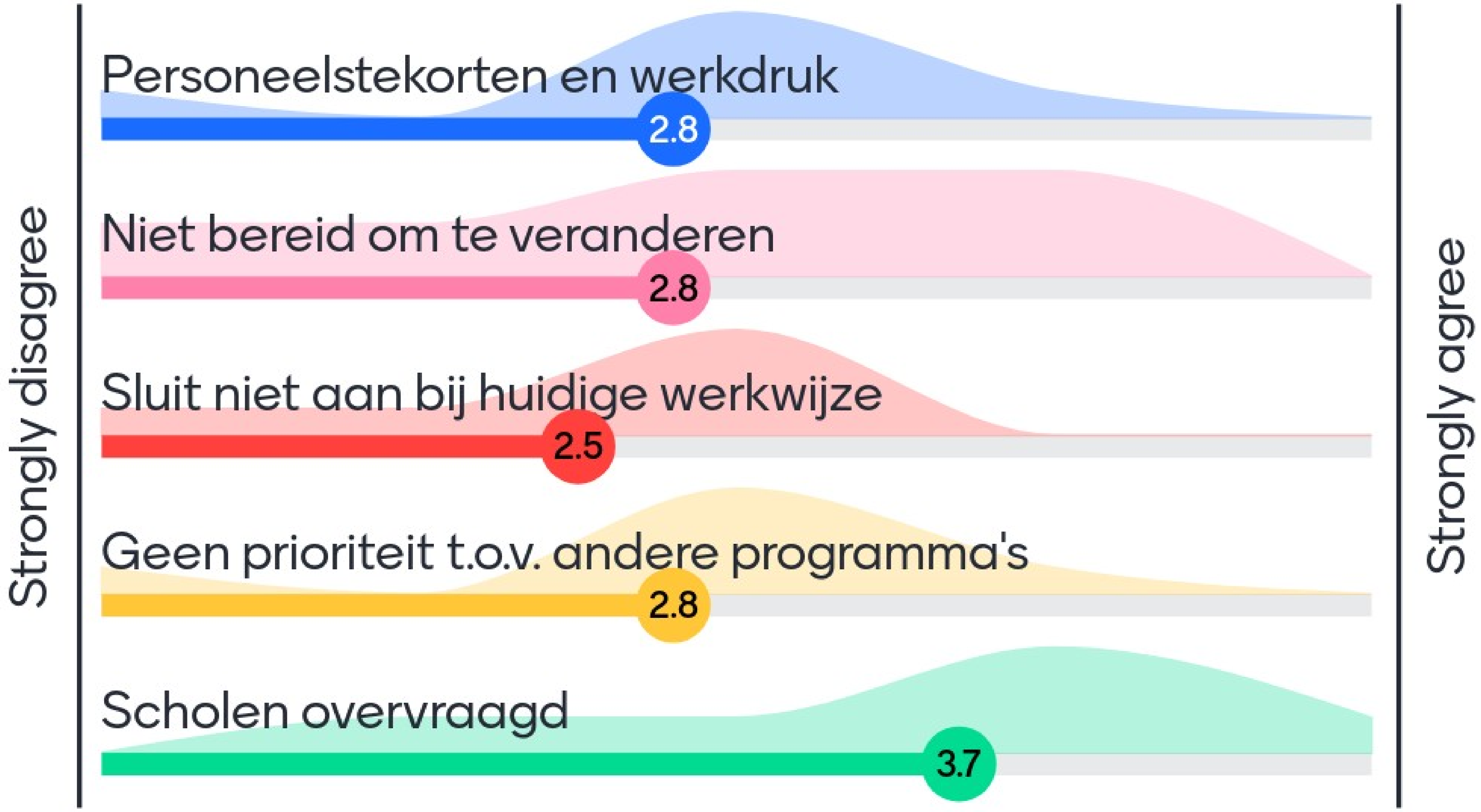

6

Mentimeter

Ik vind dat we iets moeten doen met deze belemmeringen:

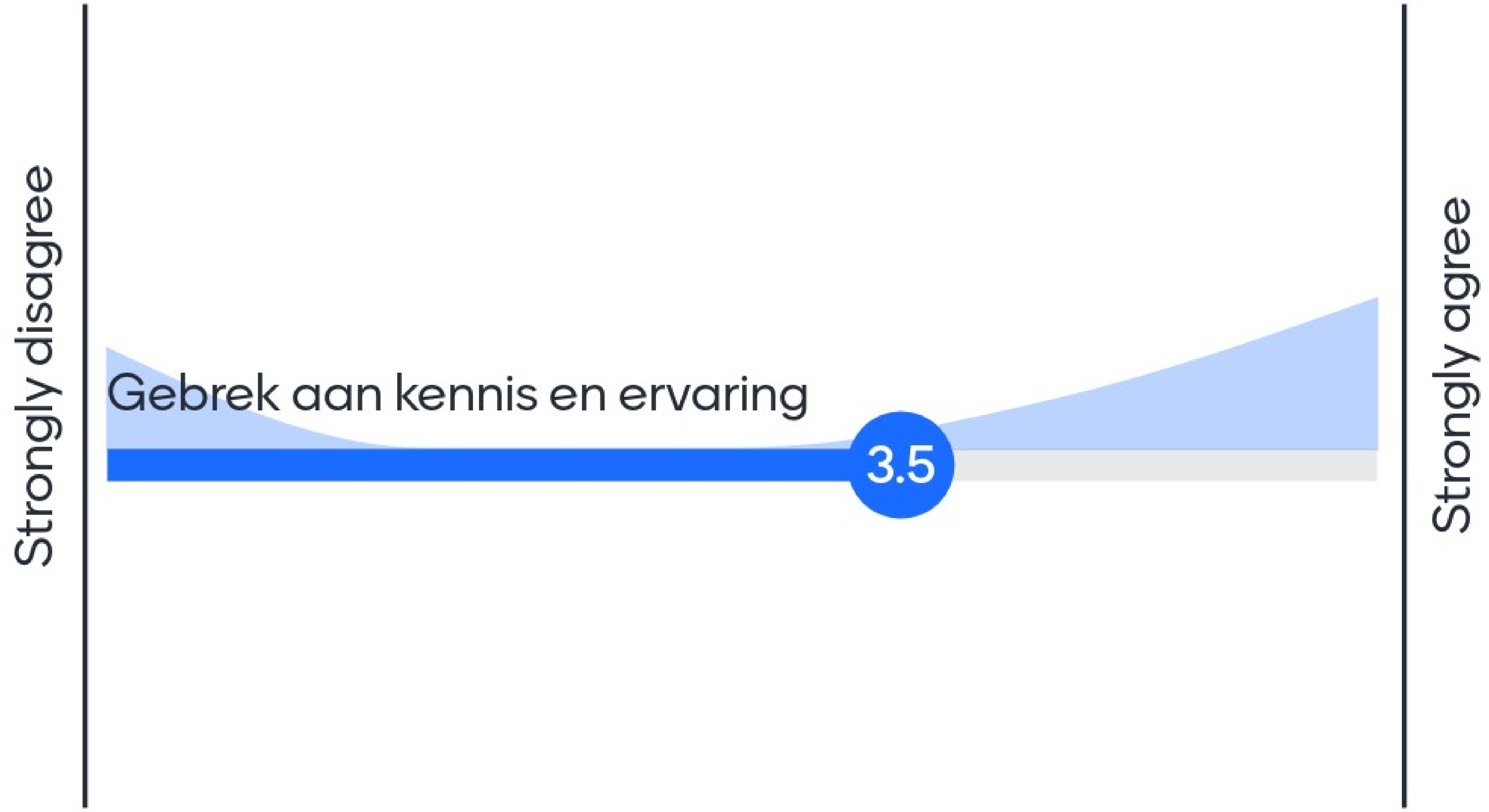

6

Ik vind dat we iets moeten doen met deze belemmeringen:

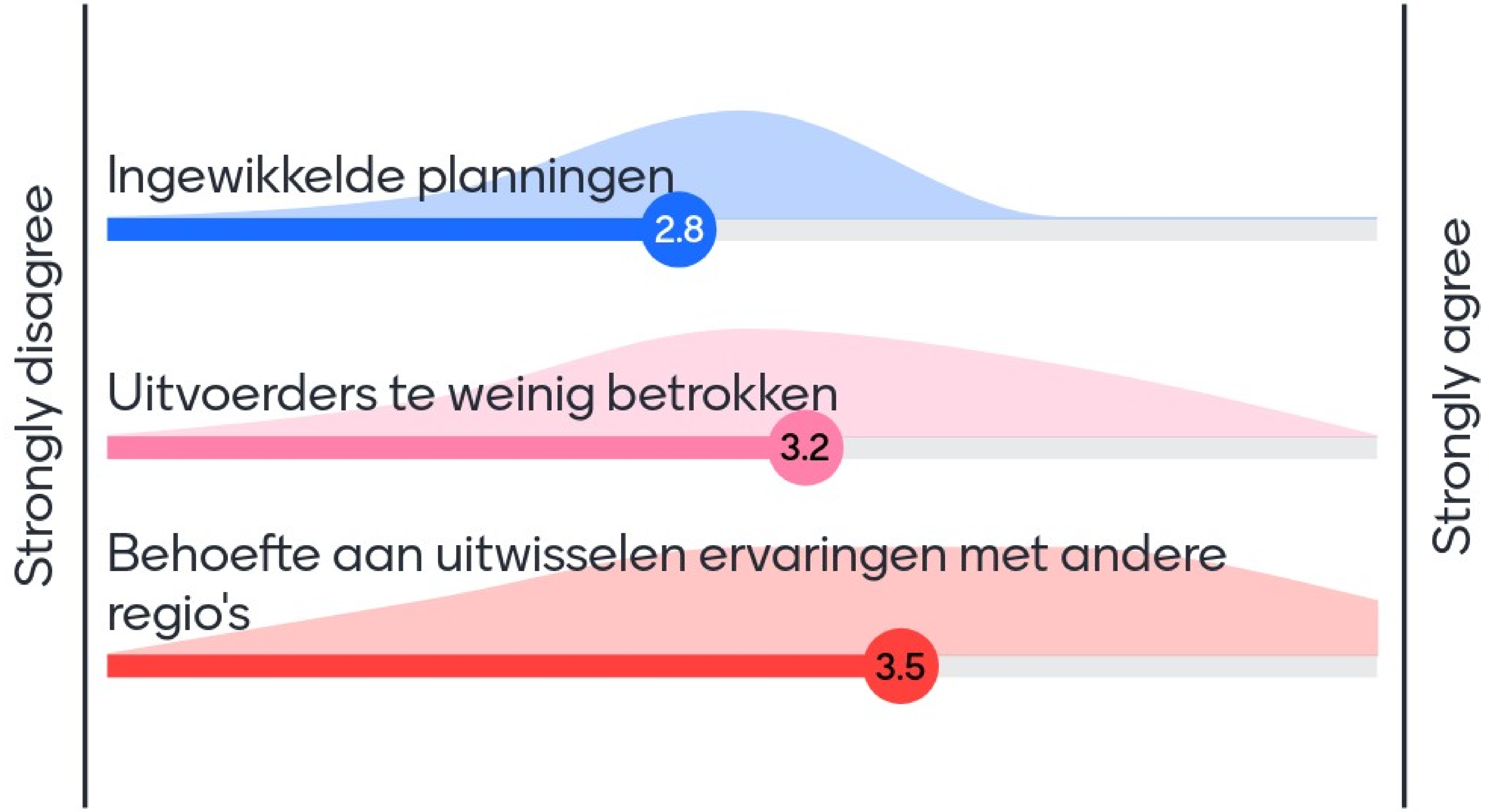

Evaluatie- en iteratieve strategieën

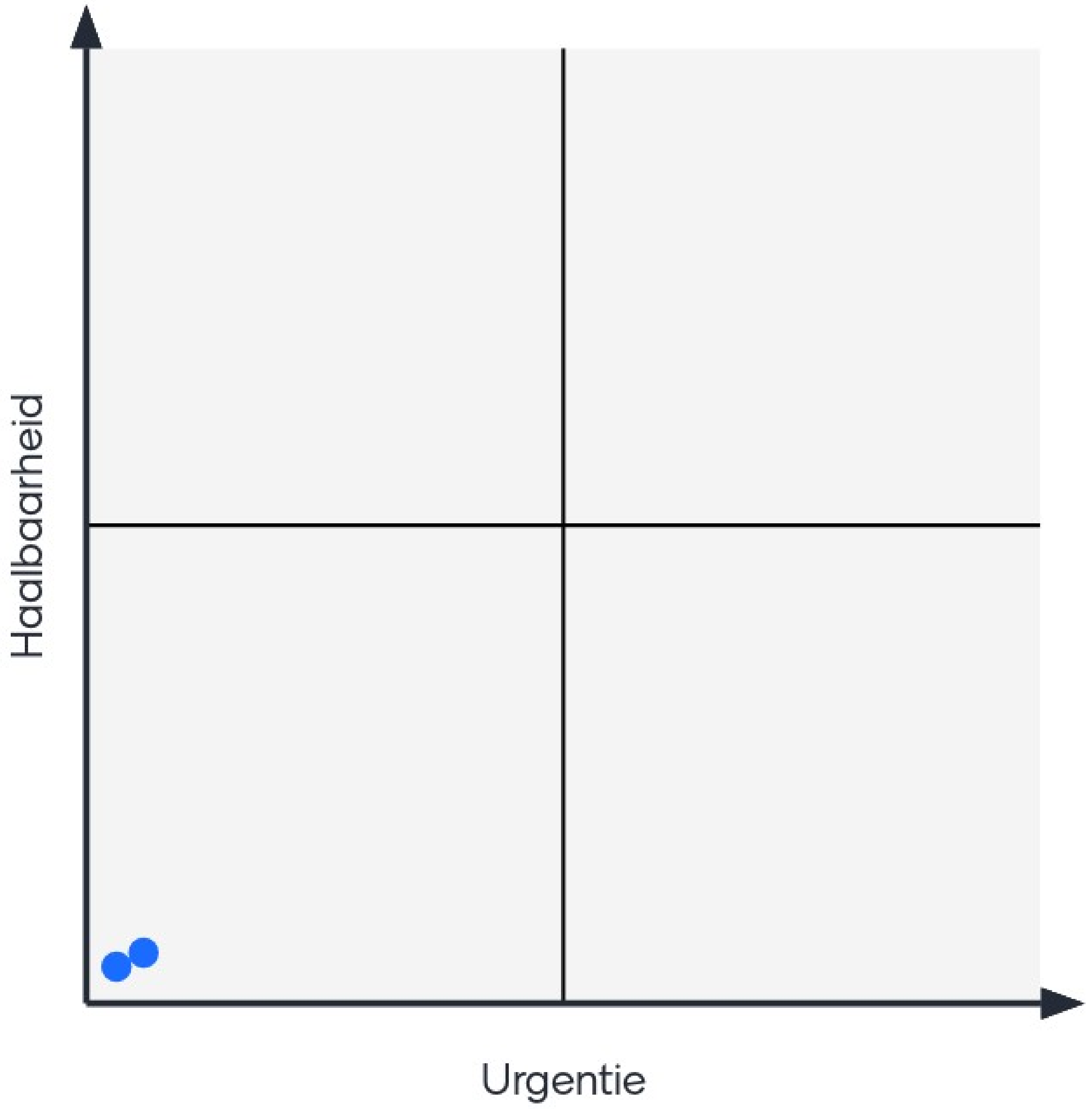

1 Stapsgewijze implementatie

Aanpassingen infrastructuur

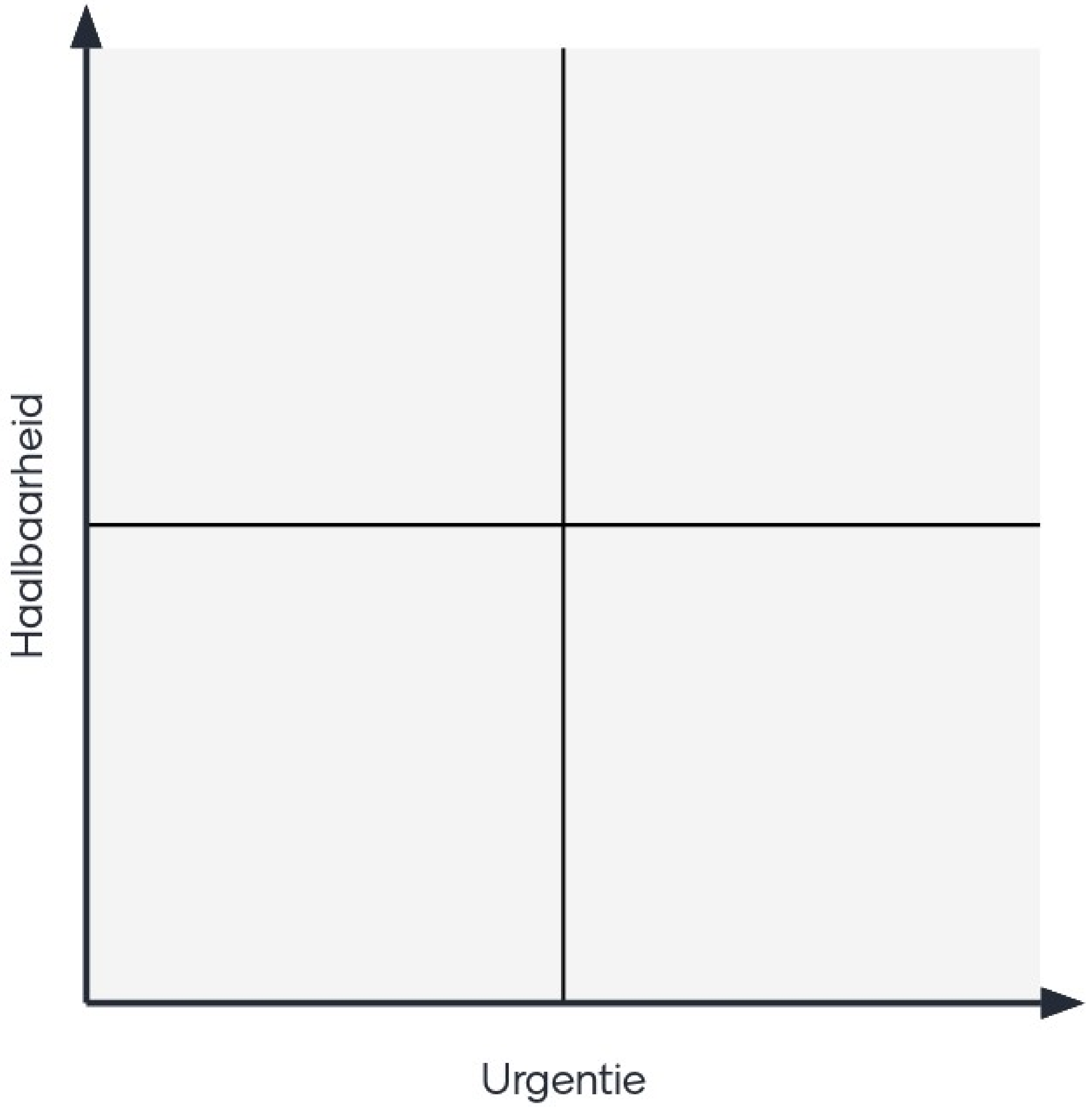

1. Minimaliseer strijdende initatieven
2. Aanpassen werkomgeving/ werkomstandigheden

Aanpassen aan context

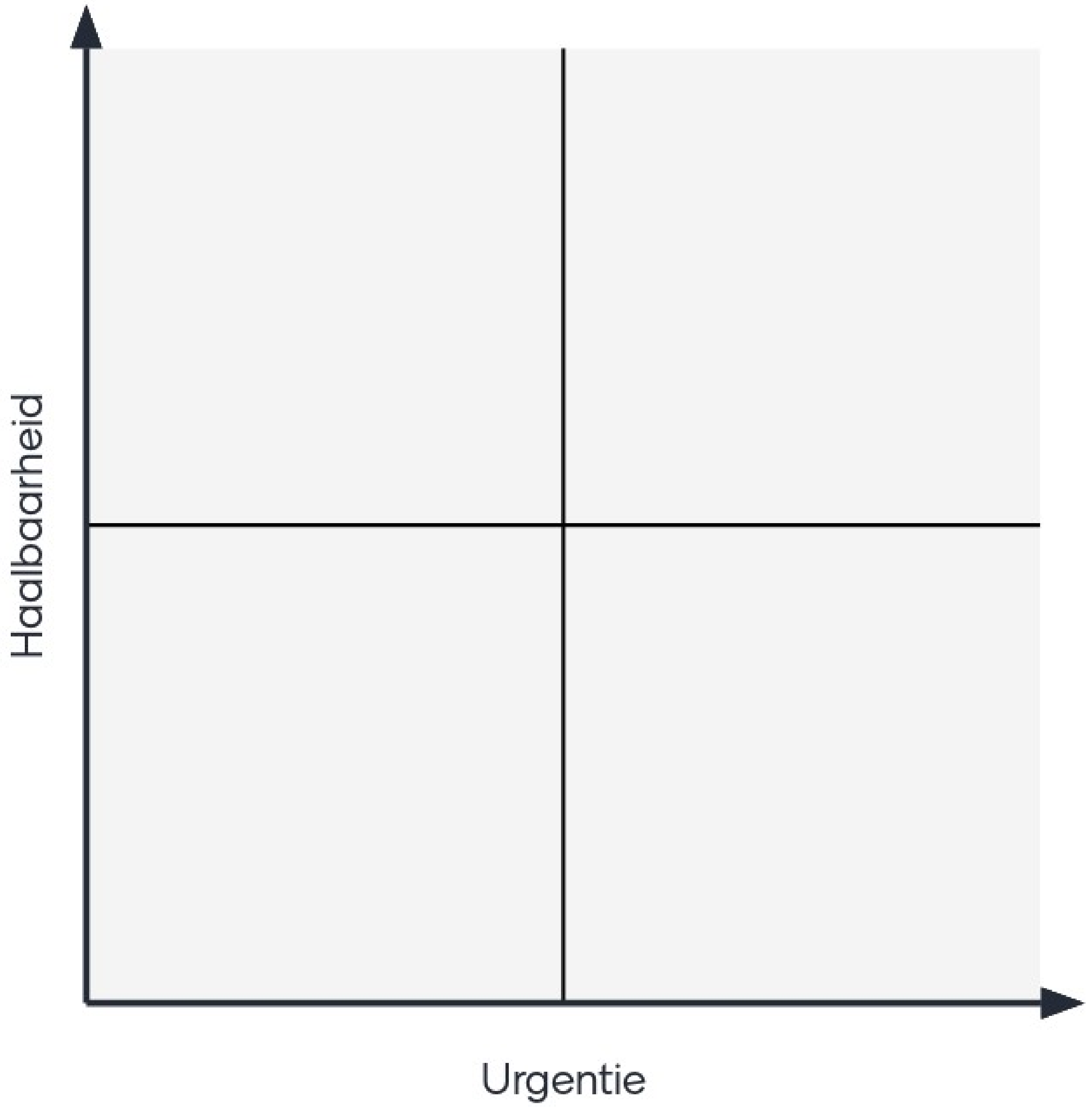

1. Bevorder aanpassen
2. Testrondes

Mentimeter

Stakeholder interrelaties

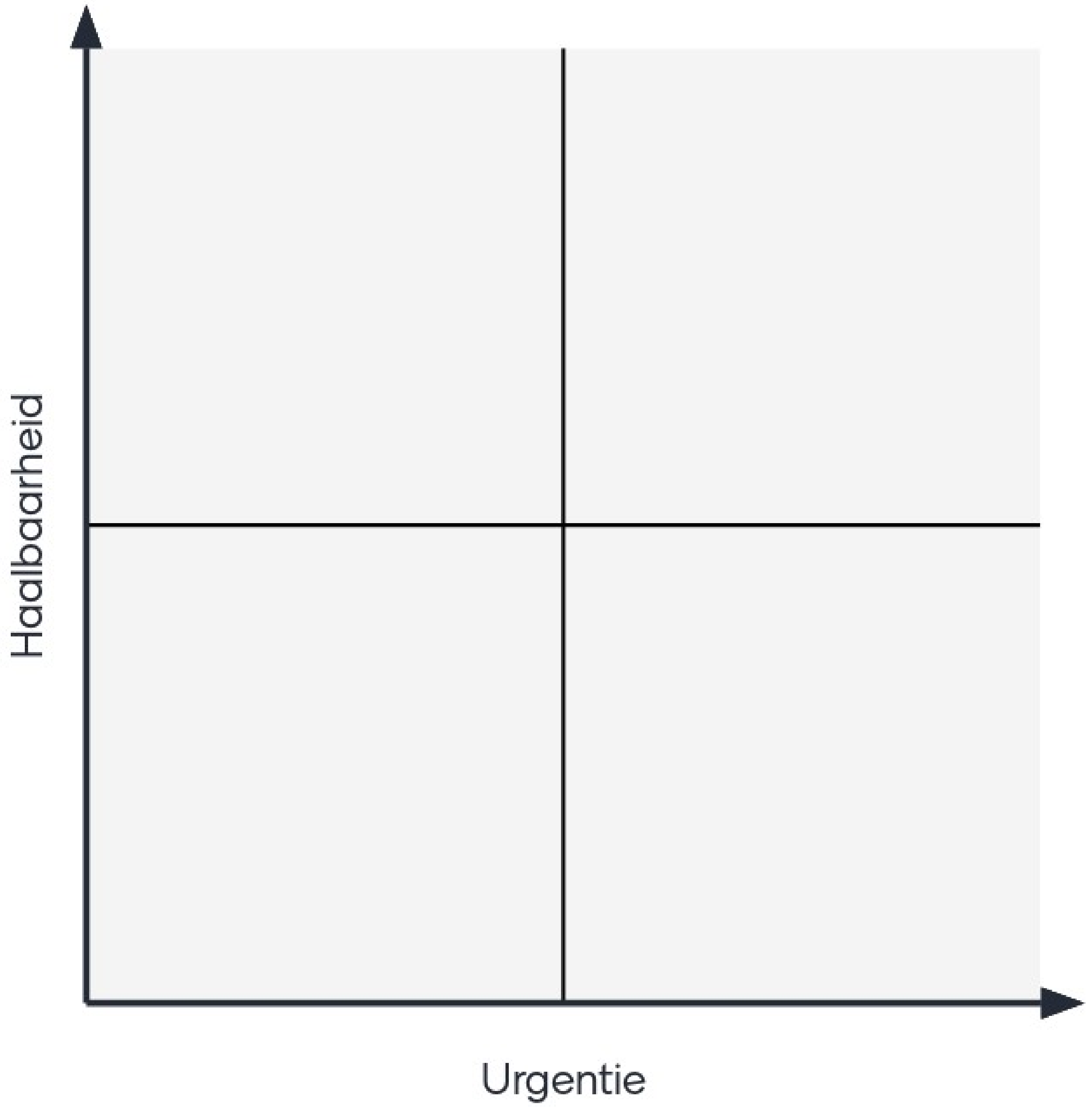

1. Vertegenwoordigers identificeren
2. Coalitie bouwen
3. Bevorder netwerkontwikkeling
4. Deel lokale kennis
5. Advies- en werkgroepen

Assisteren

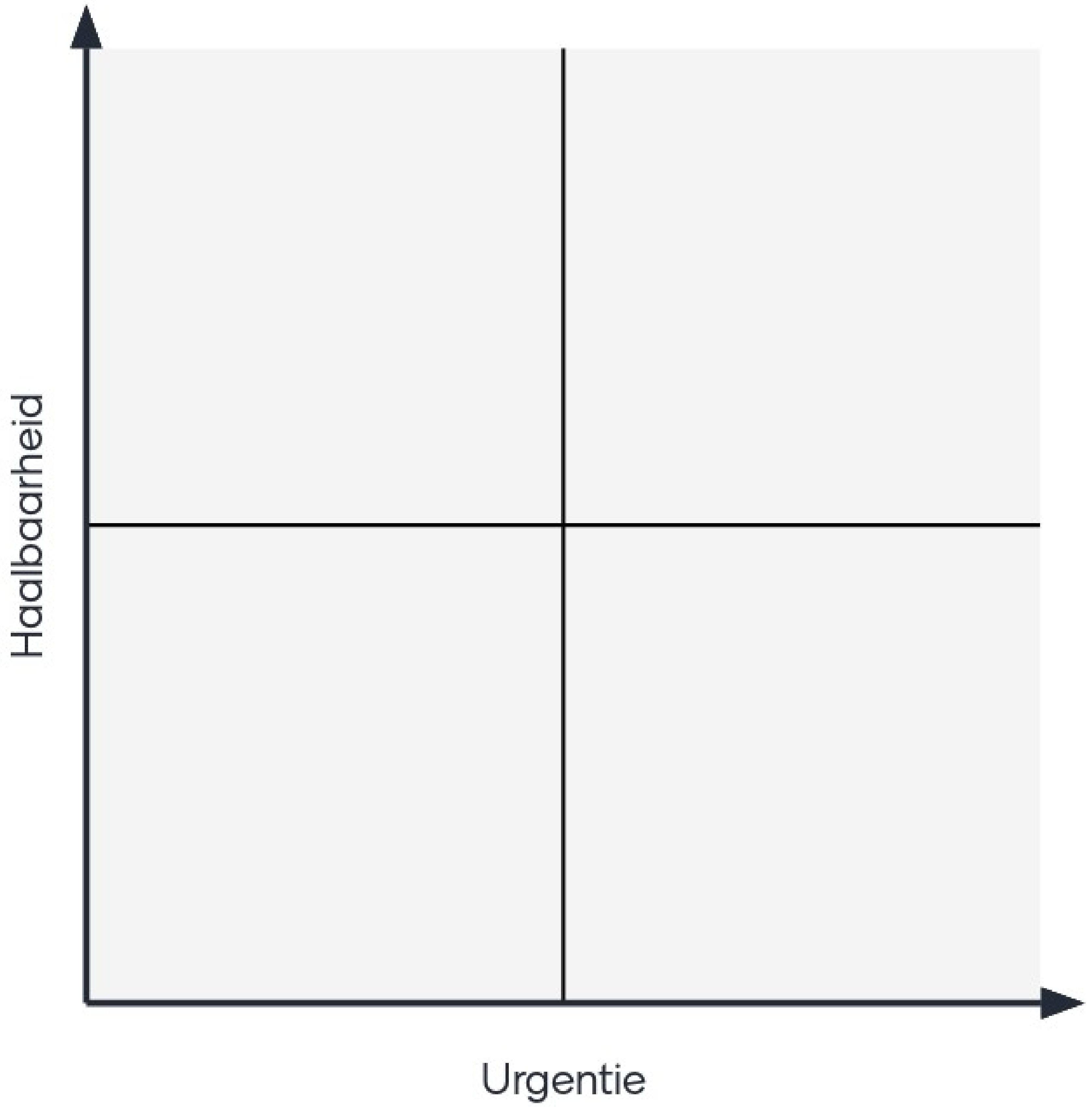

1 Facilitatie landelijk

2 Facilitatie regionaal

Mentimeter

Betrokkenen trainen en onderwijzen

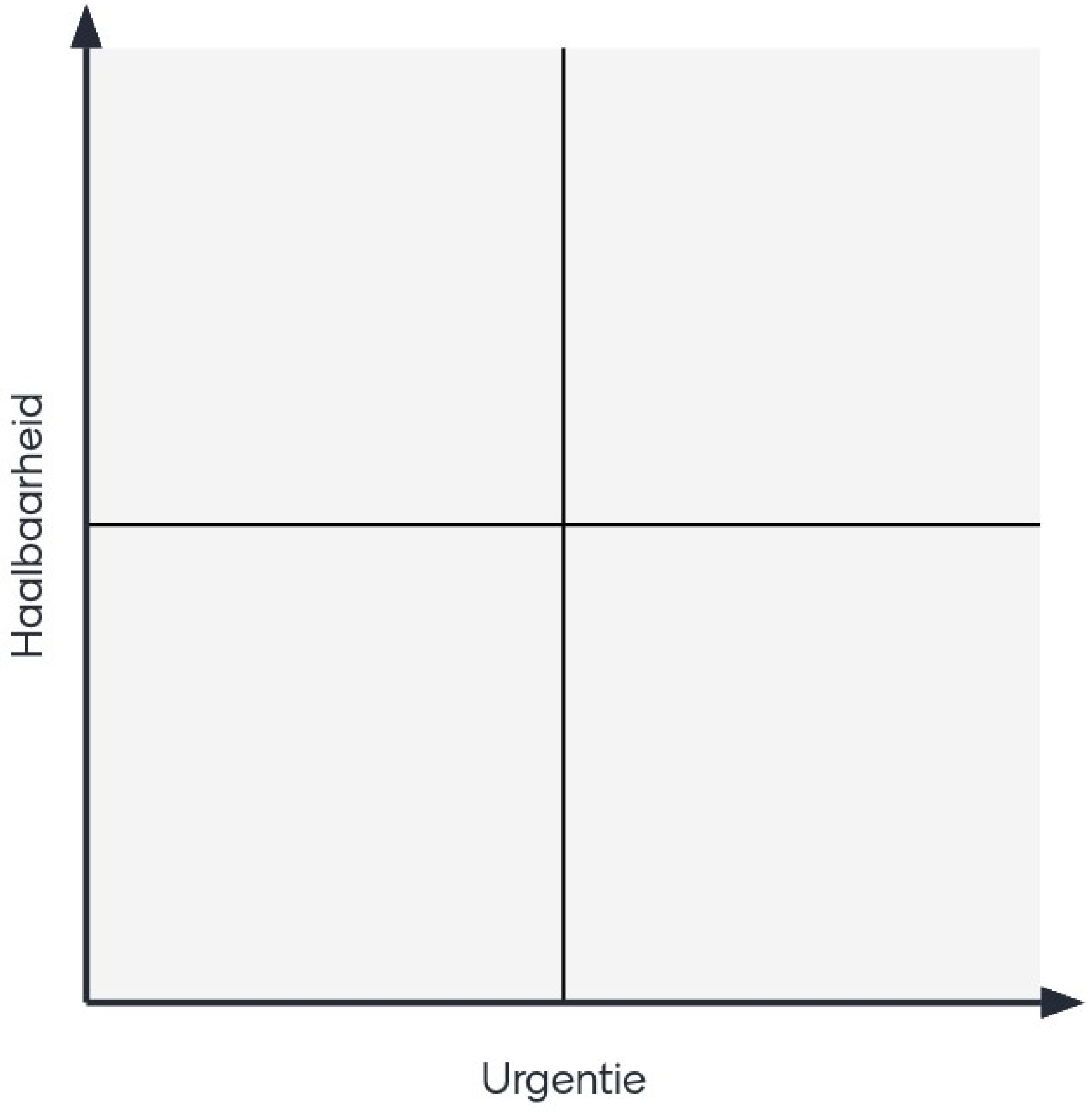

1. Ontwikkel en verspreid onderwijsmateriaal
2. Meekijken met experts
