# Supplementary material for: Development of an implementation plan for a school-based multimodal approach for depression and suicide prevention in adolescents
Source: Front Public Health. 2024 May 10;12:1386031. doi: 10.3389/fpubh.2024.1386031 (PMC11122015; doi:10.3389/fpubh.2024.1386031)
Supplement: Supplementary file 4 [file Table_3.DOCX]

| **Topic** | **Questions** |
| --- | --- |
| **Introduction** | - Could you please introduce yourself?   - Occupation and function   - Role in STORM |
| **STORM characteristics** | - What do you already know about STORM?   - *Explain STORM is needed* - What do you think about STORM?   - Fit with need for depression and suicide prevention   - Network collaboration   - Various pillars   - Complexity and feasibility - To what extent do you think STORM is suitable to implement in your region?   - Are any adaptation needed? - What is the role of your organization within STORM?   - How do you collaborate with others involved? |
| **Barriers and facilitators** | - What do you think could complicate the implementation of STORM in new regions?   - Is this currently an issue?     - What is already being done about this?   - What could complicate the implementation in the future?   - Is this specific for your region?   - Is this specific for your organization? - What do you think could facilitate the implementation of STORM in new regions?   - Is this currently an issue?     - What is already being done about this?   - What could complicate the implementation in the future?   - Is this specific for your region?   - Is this specific for your organization?   ***Follow up questions per CFIR domain if it has not been discussed yet***  *Outer setting*   - What factors from outside your organization do you think could complicate the implementation of STORM?   *Inner setting*   - How does your organization influence the implementation of STORM?     *Individuals*   - What do you expect that deliverers within your organization need to be able to work with STORM?   Or   - What do you need to be able to work with STORM? - To what extent are deliverers within your organization currently capable to work with STORM?   Or   - To what extent do you currently feel capable to work with STORM? - How do you estimate the motivation of deliverers witin your organization to work with STORM?   Or   - How motivated are you to work with STORM?   *Implementation*   - Who are currently involved in the implementation of STORM?   - How are various partners currently involved?   - What would you like to see different in the involvement of various partners?   - How do you feel about the current collaboration between those involved?   - Who else should be involved? - What plans for implementing STORM have already been made?   - What needs to happen still? |
| **Implementation goals** | - What do you think we should accomplish with the implementation of STORM? - Who do you need to achieve these goals? |
